# Spatial and temporal distribution of foot-and-mouth disease outbreaks in Algeria from 2014 to 2022

**DOI:** 10.14202/vetworld.2024.509-517

**Published:** 2024-03-05

**Authors:** Meryem Guessoum, Mounir Adnane, Ratiba Baazizi, Madina-Saliha Derguini

**Affiliations:** 1 Local Animal Resources Management Laboratory, National Veterinary School, BP161 El-Harrach, Algiers, Algeria; 2 Department of Biomedicine, Institute of Veterinary Sciences, University Ibn Khaldoun of Tiaret, Tiaret 14000, Algeria; 3 Department of Clinic, National Veterinary High School, ENSV, Algiers, Algeria; 4 Veterinary Inspection of Algiers, Department of Agricultural Services, Ministry of Agriculture, Algeria

**Keywords:** control measures, distribution, foot-and-mouth disease, livestock, outbreaks

## Abstract

**Background and Aim:**

Foot-and-mouth disease (FMD), a major transboundary animal ailment in Algeria, is a serious economic burden on the livestock sector. This study aimed to investigate the spatiotemporal distribution of FMD in Algeria and identifies the factors contributing to this phenomenon.

**Materials and Methods:**

Data on FMD cases occurring in Algeria from 2014 to 2022 were collected from various sources, including archives at the Ministry of Agriculture, peer-reviewed journal articles, conference proceedings, reference laboratory reports, and unpublished scientific reports. The data were compiled and analyzed using MS Office Excel® and SPSS® software.

**Results:**

A total of 22,690 FMD cases and 1,141 outbreaks were reported in Algeria between 2014 and 2022. The apex of infections occurred in 2014 (34.5%), followed by an increase in the number of infections in 2019 and 2017. The prevalence of FMD extended to 91.6% of the districts of the country, particularly in the north (center) and eastern regions. Cows were the most affected, with 654 outbreaks and more than 3,665 cases. Although FMD affected all four regions, there was a statistically significant long-term decline in the incidence.

**Conclusion:**

These spatial and temporal trends underscore the robust disease control methodologies implemented by the Algerian government, such as the strategic immunization of livestock to fortify their viral resistance, stringent constraints on animal mobility, and enlightenment of farmers regarding the hazards associated with unrestricted livestock movement to effectively curb FMD dissemination.

## Introduction

Foot-and-mouth disease (FMD) is a highly contagious viral infection that inflicts severe economic losses on susceptible cloven-hoofed animals, including cattle, sheep, goats, swine, and various wildlife species [1–3]. The causative agent of FMD is the *Aphthovirus* genus belonging to the *Picornaviridae* family [4]. This virus induces the formation of vesicular lesions on the feet, oral mucosa, and mammary glands, which cannot be clinically distinguished from other vesicular diseases such as vesicular stomatitis, swine vesicular disease, and vesicular exanthema of swine, all of which can exhibit comparable clinical indications [5]. The FMD virus (FMDV) exhibits antigenic diversity and includes seven recognized distinct serotypes: A, O, C, Asia-1, and South African Territories 1–3, each containing multiple subtypes and strains [6]. Although there is no cross-protection among the serotypes, there are notable serological cross-reactions [3, 7, 8].

Affected animals usually show symptoms such as fever, lameness, and vesicular lesions on the tongue, snout, feet, and teats. In severe cases, the epithelium may slough off, leading to rupture of vesicles and significant release of viral load [9]. The clinical manifestations of FMD may vary according to species, particularly in sheep, where it is particularly difficult to detect because of the lack of obvious symptoms. Unrecognized FMD infections in sheep can rapidly propagate outbreaks [10]. In most adult animals, FMD is generally not considered a fatal disease. Although it may lead to significant economic losses because of reduced productivity and trade restrictions, it does not usually lead to death in adult animals. However, FMD may be more severe in young animals, particularly in very young calves and lambs [11]. Young animals are particularly vulnerable to FMD, and the current trend in animal breeding, which focuses on efficient and disease-sensitive animals, continues to jeopardize agricultural growth in many African countries. In addition, FMD outbreaks have resulted in significant restrictions on the export of animals and animal products from affected countries [12]. The main consequences are reduced productivity, limited entry into the market, widespread culling of animal populations, adverse effects on biodiversity, and irreplaceable loss of genetic resources. The World Organization for Animal Health (OIE) has formalized an official registry of disease-free countries and regions, underlining its profound global importance [13]. FMD is not considered as a major zoonosis, however, in very rare cases, people may be infected with FMDV, usually through direct contact with infected animals or tissues. The resulting disease in humans is generally mild and self-limiting, consisting of fever and blisters on the hands and feet.

FMD is considered an endemic disease in specific regions worldwide, affecting extensive areas of Africa, Asia, and South America. The geographical distribution of each FMD serotype varies, with serotype O having the widest spread [14, 15]. Direct losses caused by FMD, such as reduced milk and meat production, and indirect losses significantly impact the food security and livelihoods of communities in low-income areas where FMD is prevalent [3, 13, 16, 17].

After the recurrence of FMD in Algeria in 2014, FMD continued to be a persistent problem, and Algeria has been designated as an FMD-afflicted region. Health authorities work diligently to eradicate the disease, and breeders, veterinarians, and other experts serve as the first line of defense in the detection chain. To mitigate the risk of the introduction and spread of FMD, veterinary services have implemented vaccination of cattle. Prophylactic measures included the control of animal movements, vaccination in farms and livestock markets, and strict control in slaughterhouses. These efforts have significantly reduced the incidence of FMD in Algeria. The national control plan recommended by the Organization for Economic Cooperation and Development (OIE) serves as a strategy for FMD management in Algeria. This approach requires a comprehensive understanding of the spatiotemporal distribution of FMD, which involves analyzing the monthly, yearly, and geographical clusters of FMD outbreaks.

This study aimed to describe the epidemiology of FMD in Algeria from 2014 to 2022, with a particular focus on evaluating the effectiveness of the control measures implemented.

## Materials and Methods

### Ethics approval

Ethics approval was not applicable to this study.

### Study period and location

The data were collected for FMD outbreaks in Algeria from 2014 to 2022. Algeria, located in North Africa, is the largest country on the African continent and the tenth largest in the world. The border connects Tunisia to the northeast, Libya to the east, Niger to the southeast, Mali to the southwest, Mauritania and Western Sahara to the west, and Morocco to the northwest. To the north, Algeria has a coastline along the Mediterranean Sea. With an area of 2.3 million km^2^, Algeria is characterized by a diverse geographical landscape, ranging from coastal areas along the Mediterranean coast to vast desert areas in the south.

The distribution of livestock production systems in Algeria varies significantly due to the country’s large geographical area, resulting in different weather patterns in different regions, where small ruminants are predominantly concentrated in the steppes and dairy farms are more prevalent in coastal regions [18, 19]. The livestock sector contributes approximately 5%–10% of the total income of farmers, predominantly smallholders who represent 70% of the livestock production in Algeria [20]. These small farms in Algeria usually lack awareness about biosecurity measures, leading to unrestricted movement of animals. In addition, limited access to information on livestock management and veterinary services is considered a significant constraint for livestock production activities.

National FMD surveillance data from 2014 to 2022 were obtained from the Department of Animal Health (DAH) in the Algerian Ministry of Agriculture and Rural Development (MADR). These national data contain information on the number of cases, animal species (cattle, goats, and sheep), identified serotypes, and estimated outbreak dates and locations in communes, districts, and municipalities. The data were collected by the local sub-DAH through passive surveillance on a daily/weekly basis. Most reported cases were diagnosed on the basis of clinical observations by local animal health professionals, whereas some cases were confirmed through laboratory tests performed at the national level.

### Data collection

Algeria is geographically divided into 48 wilayas, which are equivalent to administrative provinces or territorial communities. The country is divided into four main administrative regions: central, western, eastern, and northern. These regions are further divided into 160 sub-prefectures and 1540 communes. Note that Algeria underwent a reorganization in 2021, which resulted in the establishment of 58 departments. However, it should be noted that this new distribution in statistical services is yet to be fully implemented.

This study used a retrospective case series approach to examine FMD. The data for this study were collected from MADR and the Algerian Ministry of Public Health (MPH). FMD was defined based on clinical observation of characteristic lesions and confirmed through laboratory analysis.

Data on FMD cases in Algeria between 2014 and 2022 were collected from different sources. These include peer-reviewed journal articles, conference proceedings, reference laboratory reports, unpublished scientific reports, and gray literature. The gray literature encompassed records from authoritative sources such as the National Epidemiology Unit, the Central Veterinary Research Laboratory’s Virology Database, annual reports from the Ministry of Agriculture, and FMD reports from the National Institute of Animal Health.

The case data consisted of details such as the date of occurrence, location, number of cases, population at risk (if available), serotype involved, type of vaccination, and type of vaccine used. Subsequently, outbreak information and livestock data were recorded in Microsoft Excel 2010 (Microsoft Office, Washington, USA) and analyzed using STATA version 14 software (StataCorp. TX, USA).

### Exclusion and inclusion criteria

Only FMD outbreaks in cattle, sheep, and goats were identified as inclusion criteria in this study. This information was limited to outbreaks occurred from July 2014 to December 2022.

### Statistical analysis

MS Office Excel® software was used to compile and analyze data on the number of FMD cases and outbreaks. Continuous data are presented as averages, and categorical variables are presented as frequencies and percentages. The SPSS® Statistics Version 20.0 (IBM Corp., NY, USA) enabled the use of a 5% confidence interval and Chi-square test. The difference was significant when the p-value was 5%.

## Results

### Case distribution of FMD infections by years

**Figure-1:**
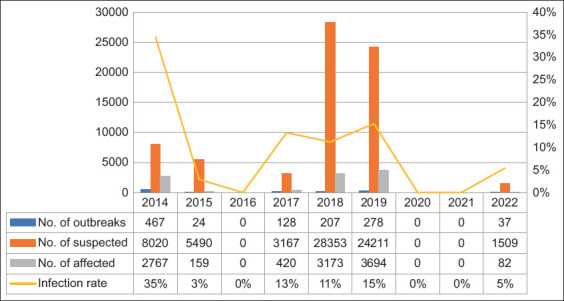
Annual foot-and-mouth disease outbreaks, suspected cases, and affected cases recorded in Algeria between 2014 and 2022. N°= number.

**Figure-2:**
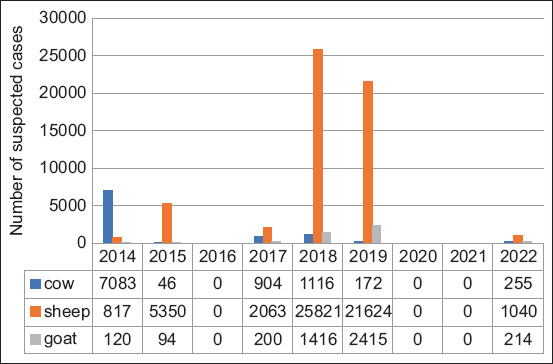
Annual distribution of foot-and-mouth disease cases reported in Algeria, by species, during 2014–2022.

From 2014 to 2022, there were 70,750 cases of FMD in all 48 departments of Algeria. The highest infection rate was observed in 2014 (p < 0.05), accounting for 34.5% of cases, followed by 2019 (15.3%), 2017 (13.3%), and 2018 (11.2%). In contrast, 2015 and 2022 exhibited the lowest infection rates of 2.9% and 5.4%, respectively (Figure-1). Of these, 80.1% (56,715 cases), 13.5% (9,592 cases), and 6.3% (459 cases) were reported in sheep, cattle, and goats, respectively (Figure-2).

### Temporal distributions by years of FMD outbreaks

**Figure-3:**
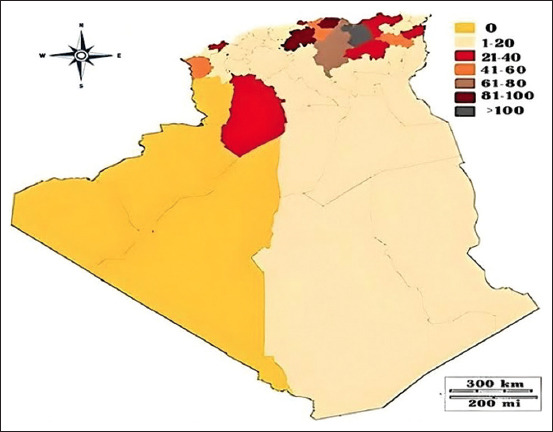
Spatial distribution of foot-and-mouth disease in the districts of Algeria (2014–2022). The map depicts the district-level administration divisions represented by gray lines. The shaded area highlights the cumulative reports of foot-and-mouth disease outbreaks in each district from 2014 to 2022, providing an overview of the disease’s distribution across the country over the specified period [Source: The base map was adopted from https://www. pngegg.com/en/png-chhsm, accessed November 5, 2023].

From July 2014 to the end of 2022, 1,141 FMD outbreaks were recorded in Algeria. These outbreaks occurred in 91.6% (44 out of 48) districts of the country (Figure-3). The highest number of outbreaks (p = 0.001) was reported in 2014 (40.9%), followed by 2019 (24.1%). On the other hand, 2015 exhibited the lowest outbreaks at 2.1% and 2022 at 3.5% (Figure-1).

### Regional distributions and temporal patterns of FMD cases

During the study period, the northern (center) region reported the highest number of outbreaks (460 outbreaks; 40.3%), followed by the eastern region (382 outbreaks; 33.4%) (Figure-3). The southern and western regions had the lowest number of outbreaks, with 156 (13.6%) and 143 (12.5%) outbreaks, respectively.

**Figure-4:**
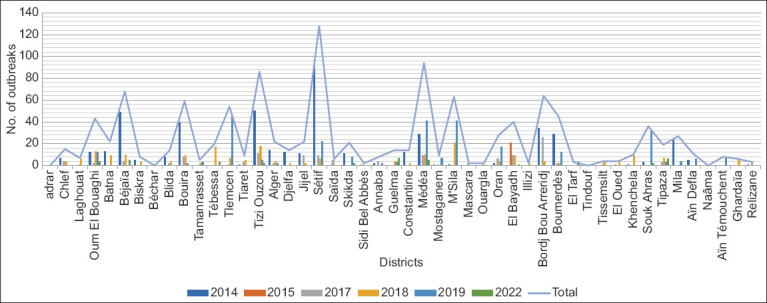
Spatial distribution of foot-and-mouth disease in Algerian districts during 2014–2022.

**Figure-5:**
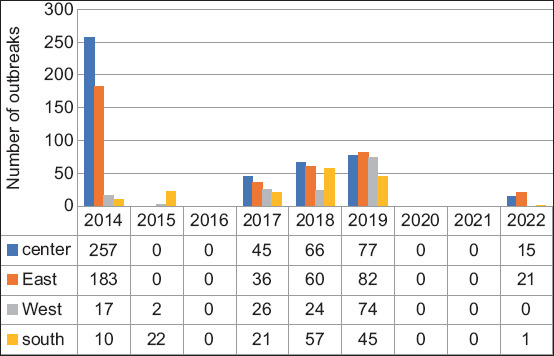
Distribution of foot-and-mouth disease outbreaks in Algerian regions and per year during 2014–2022.

Significant regional variations were observed in the number of reported outbreaks within the same year (p = 0.05) (Figure-4). In 2014, 257 cases (55%) occurred in the central region, followed by 183 cases (38.7%) in the eastern region. Similar trends emerged in 2017 and 2018, with the central and eastern regions being the most affected regions. In 2015, the southern region experienced the highest outbreak rate (91.6%). Notably, in 2019 and 2022, the eastern region faced the highest impact, with 82 outbreaks (29.4%) and 21 outbreaks (56.7%), respectively (Figures-4 and 5).

In 2014, the highest incidence of infection was observed in cattle, with 2,755 of 7,083 cows showing clinical symptoms in the outbreak regions (Figure-2). A cumulative incidence of 1,009 affected cows was observed in the district of Setif in the northeastern part of the country (Figure-4). Adjacent areas such as Tizi-Ouzou, Bejaia, Mila, and Bouira also reported varying infection rates (Figure-3). Suspected cases of FMD were observed in 200 goats and 1,572 sheep, with the El-Bayadh region reporting the highest infection rate among sheep, affecting 142 animals.

In 2017, 128 FMD outbreaks were detected in different regions, with distribution as follows: 35% (45/128) in the central region, 28% (36/128) in the east, 20% (21/128) in the west, and 16% (21/128) in the south. Of the 420 confirmed cases, 59.3% (249 cases) were observed in cattle, whereas 40.7% (171 cases) were identified in small ruminants. In 2018, the majority of FMD outbreaks occurred in the northern regions of the country, with some outbreaks also reported in the western and southeastern regions. Overall, a total of 207 FMD outbreaks affected 580 ruminants.

In 2019, 278 FMD outbreaks were reported. The highest number of outbreaks occurred in the eastern region (29.5%), followed closely by the central and western regions. Small ruminants showed the highest susceptibility, with 24,039 clinically diagnosed cases.

In 2022, there were 37 reported FMD outbreaks. Out of 1,509 suspected cases, 82 were confirmed, with 80 cases affecting cattle. Table-1 summarizes FMD outbreaks and cases from 2014 to 2022.

**Table-1:** Annual outbreaks of foot-and-mouth disease, suspected cases, affected cases, animal species and regional occurrence in Algeria (2014–2022).

Year	Animal species	Region	Number of outbreaks	Number of suspected cases	Number of affected animals	Infection rate (%)
2014	Cow	Center	238	2620	1161	44.3
		East	155	4050	1458	36.0
		West	15	372	115	30.9
		South	7	41	21	51.2
	Sheep	Center	12	279	12	4.3
		East	18	531	0	0.0
		West	1	1	0	0.0
		South	1	6	0	0.0
	Goat	Center	7	73	0	0.0
		East	10	39	0	0.0
		West	1	1	0	0.0
		South	2	7	0	0.0
2015	Cow	Center	0	0	0	0.0
		East	0	0	0	0.0
		West	0	0	0	0.0
		South	5	46	1	2.2
	Sheep	Center	0	0	0	0.0
		East	0	0	0	0.0
		West	2	460	16	3.5
		South	10	4890	142	2.9
	Goat	Center	0	0	0	0.0
		East	0	0	0	0.0
		West	0	0	0	0.0
		South	7	94	0	0.0
2017	Cow	Center	32	127	0	0.0
		East	32	302	95	31.5
		West	22	401	136	33.9
		South	14	74	18	24.3
	Sheep	Center	9	575	17	3.0
		East	3	60	2	3.3
		West	4	716	4	0.6
		South	4	712	132	18.5
	Goat	Center	4	42	2	4.8
		East	1	8	0	0.0
		West	0	0	0	0.0
		South	3	150	14	9.3
2018	Cow	Center	50	423	327	77.3
		East	23	414	143	34.5
		West	11	116	34	29.3
		South	9	163	76	46.6
	Sheep	Center	12	1095	81	7.4
		East	26	10219	780	7.6
		West	10	2205	266	12.1
		South	30	12302	1405	11.4
	Goat	Center	4	87	18	20.7
		East	11	531	6	1.1
		West	3	93	2	2.2
		South	18	705	35	5.0
2019	Cow	Center	5	8	0	0.0
		East	8	56	0	0.0
		West	6	100	0	0.0
		South	3	8	0	0.0
	Sheep	Center	49	3683	628	17.1
		East	46	7355	1055	14.3
		West	42	6746	1107	16.4
		South	27	3840	617	16.1
	Goat	Center	23	394	96	24.4
		East	28	790	79	10.0
		West	26	986	82	8.3
		South	15	245	30	12.2
2022	Cow	Center	11	114	72	63.2
		East	8	141	8	5.7
		West	0	0	0	0.0
		South	0	0	0	0.0
	Sheep	Center	2	16	0	0.0
		East	8	989	0	0.0
		West	0	0	0	0.0
		South	1	35	2	5.7
	Goat	Center	2	4	0	0.0
		East	5	210	0	0.0
		West	0	0	0	0.0
		South	0	0	0	0.0

## Discussion

This study identifies the temporal and spatial patterns of FMD in Algeria from 2014 to 2022. The disease remained prevalent throughout the study period, with notable peaks in 2014, 2018, and 2019. Algeria is listed as an FMD-endemic country, and the disease continues to pose economic and productivity challenges [21]. During the study period, 1,141 outbreaks were recorded, with an average annual incidence of 138.8 outbreaks. The distribution of outbreaks was extensive, covering almost all regions, with a higher incidence in the central and eastern regions.

In 1999, there was a call for increased alertness in all Algerian regions, leading to a comprehensive active monitoring of farms. All veterinary professionals were mobilized and strictly adhered to the biosafety measures. Media channels have been used to raise awareness, emphasizing farmers’ participation in disease prevention and control programs to protect livestock. All cattle affected on the farms were culled, and the owners received appropriate compensation from the local government. In addition, enhanced surveillance activities covered a 10-km radius of the outbreak zone. Extensive vaccination campaigns included 1.4 million cattle and 160,551 boosters every month. In addition, 600,000 sheep and 34,733 goats were vaccinated around the outbreak areas. Since 1999, Algeria has reported no FMD cases [22].

On July 23, 2014, an FMD outbreak was detected in the Setif district, eastern Algeria, near the Tunisian border [23, 24]. The outbreak originated on a cattle farm and was linked to the illegal introduction of animals from Tunisia. Subsequent testing identified the virus as part of the O/MESA/Ind2001d lineage, which is closely related to strains isolated during Tunisia’s 2014 FMD outbreaks [22]. The outbreak spreads rapidly, and cases were documented in many districts. Since late August, more than 350 outbreaks have been officially registered in 33 different districts. Notably, none of the documented cases involved cattle, with no clinical or serological evidence of FMD in small ruminants.

To limit the spread of the disease, regional authorities have put in place a national emergency vaccination program targeting cattle. In addition, imports of susceptible animal species from high-risk countries have been temporarily suspended. Rigorous sanitary measures, such as the isolation of infected animals, hygienic slaughter procedures, and thorough disinfection, have been rigorously enforced within households and their surroundings. These comprehensive measures effectively halt the transmission of the disease and prevent the emergence of new cases.

In 2015, there was a significant decrease in the number of recorded outbreaks, which could be attributed to extensive vaccination campaigns initiated in response to the 2014 outbreaks. Together with the early detection and culling of affected animals, these efforts are likely to have contributed to the decline in reported cases. Notably, a significant number of cases have been reported in western Algeria, coincident with the official outbreak of FMD in Morocco, involving the same serovar (O) [25].

According to the literature, sheep are typically asymptomatic or exhibit mild symptoms of FMD, yet they play a key role in transmitting the virus to more vulnerable ruminants, especially cattle [26]. However, some studies have indicated that sheep can also develop clinical signs of FMD because of the high mutation rate of the virus [27]. The absence of FMD outbreaks reported in Algeria in 2016 suggests the effectiveness of the control measures implemented after the 2014–2015 epidemic.

In 2017, Algeria reported a new outbreak of FMD associated with a different strain, specifically serotype A. Four outbreaks associated with FMD serotype A occurred in the northern part of the country [28]. Subsequently, virus samples were forwarded to the OIE reference laboratory in Brescia, Italy (IZSLER), for confirmation and genetic lineage identification through sequence analysis. Phylogenetic analysis focusing on the VP1 coding gene revealed that FMDV serotype A detected in Algeria belonged to the A/Africa topotype, specifically falling within lineage G-IV. This lineage is endemic to sub-Saharan countries. Genetic analysis revealed that the closest genetic relatives of this Algerian serotype A virus are type A isolates from Nigeria dating back to 2015.

The FMD World Reference Laboratory at the Pirbright Institute conducted vaccine-matching tests to identify the most suitable vaccine strain for potential use against a specific identified strain. According to the 2017 Biennial Report submitted by the Algerian authorities, a significant preventive vaccination campaign was conducted between July and December 2017. This large-scale vaccination campaign targeted almost two million cattle and involved the administration of a bivalent vaccine intended to confer immunity against serotypes A and O. However, the report did not specify the effectiveness of this vaccine against the previously identified strain. There is a need for further clarification and research to determine the efficacy of the vaccine against these specific FMD strains.

Since July 9, 2018, Algerian veterinary authorities have reported a worrying resurgence of FMD, with nine new outbreaks documented across various regions in the northern part of the country. In response, many strict control measures have been swiftly implemented, including the culling of infected animals, strict restrictions on livestock movement, thorough disinfection of affected farms, and establishment of strict surveillance protocols both within and outside designated containment and protection zones [29]. Serotype O, belonging to the East Africa 3 topotype, was the FMD strain identified in these outbreaks [29]. Notably, FMD exhibited rapid transmission, with 207 FMD outbreaks detected. The Tizi-Ouzou region emerged as the epicenter of this outbreak, which was characterized by an alarmingly elevated infection rate. In this region, cattle species were the most affected by the disease, with an average infection rate of 49.5%. In contrast, sheep and goats exhibited infection rates of 10.1% and 7.7%, respectively.

Tizi-Ouzou, known for significant dairy production in Algeria, witnessed a notable spread of FMDV, potentially attributed to livestock trade within animal market. FMDV, characterized by rapid replication within infected animals and transmission to susceptible animals in close proximity, including through aerosol transmission, likely contributed to the extensive outbreak in Tizi-Ouzou [1]. As in the case of previous outbreaks, immediate vaccination campaigns targeting cattle with a bivalent vaccine containing serotypes A and O were launched following the identification of this disease. Temporary closure of livestock markets has been conducted, and newly acquired animals have been placed under quarantine as part of the control measures.

In 2019, Algeria reported 278 FMD outbreaks with no clinical infection in bovine species; however, 3,694 confirmed cases occurred in sheep. In addition, there was a relatively high incidence of infection among goats, affecting 287 animals. These findings are mainly based on comprehensive veterinary control measures targeting cattle populations across multiple wilayas. All essential preventive measures have been rigorously enforced to prevent this epizootic disease and minimize the risk of livestock infection. Despite the stringent implementation of preventive measures and directives regarding the culling of infected animals, resistance persists among some farmers, presenting a challenge to containment efforts.

Inspection teams have successfully mobilized all necessary human and material resources to effectively control disease outbreaks, with particular emphasis on enclaves and border regions. To ensure effective control of animal health in the region, officials have called for involving relevant authorities and associations. Coordination meetings were held with livestock breeders to assess herd sensitivity and locations, strategize vaccination campaigns, outline preventive measures necessary to protect animal health in border areas, and coordinate the mobilization of mixed brigades in cooperation with Saharawi refugees to vaccinate valuable animals.

Algeria remained free from reported FMD outbreaks in 2020 and 2021, underscoring the continued effectiveness of comprehensive FMD control measures implemented in response to the 2018–2019 outbreaks. However, a resurgence occurred in 2022, with 37 outbreaks reported in nine provinces. Among ruminant species, bovines exhibited the highest average infection rate, emphasizing the persistent need for vigilance and effective FMD control strategies.

The recurrence of FMD outbreaks in Algeria can be attributed to different factors. Insufficient vaccination coverage in susceptible animal populations is a significant contributor to FMD outbreak recurrence [30]. Challenges, such as inadequate vaccination campaigns and difficulties in reaching remote areas, can lead to the existence of pockets of vulnerable animals [31], highlighting the need for comprehensive vaccination programs. In addition, the persistence of FMDV in the environment, particularly in contaminated equipment, clothing, or vehicles, can introduce the virus to new areas or herds even after initial control [32]. This highlights the need for strict biosecurity measures to prevent virus transmission through fomites and other environmental sources.

In addition, wild animals, especially cloven-hoofed species, can sometimes act as reservoirs for FMDV [33], emphasizing their role in carrying and transmitting the virus to domestic livestock. Movement of infected animals or contaminated products between regions can facilitate the geographical spread of FMD. Moreover, considerations related to the virus itself are crucial. Recent research indicates that FMD virus can undergo genetic changes, potentially leading to the emergence of new strains or serotypes [34]. These genetic variants may not be effectively covered by existing vaccines, leading to recurrence. To address the evolving nature of the virus, ongoing surveillance and vaccine development efforts are essential.

In addition, favorable temperature and humidity conditions create an environment conducive to the survival and transmission of the FMDV [35]. These conditions can enhance the persistence of the virus in the environment, potentially leading to increased transmission. Furthermore, human activities such as handling infected animals, improper disposal of carcasses, and accidental virus release from laboratories contribute to the recurrence of FMD outbreaks. These activities pose a risk of introducing the virus into new areas or populations and highlight the need for strict biosecurity measures and responsible practices to mitigate these risks.

## Conclusion

The temporal distribution of FMD in Algeria from 2014 to 2022 showed significant variability, which was influenced by factors such as inadequate control measures, difficulties in regulating livestock movements, changes in diagnostic procedures, and free trade policies. In view of the significant economic impact and transboundary nature of the disease, these findings highlight the need for the Algerian government to strengthen regional control programs and surveillance systems. To effectively combat FMD in Algeria, a multifaceted approach is recommended. First, comprehensive education initiatives for cattle farmers should focus on the benefits of vaccination and active participation in control programs. Concurrently, consider the re-establishment of quarantine stations to monitor the movement of cattle and prevent disease spread. In addition, significant investment in the development of a novel vaccine offering long-term protection and reduced post-vaccination responses is crucial. This technological advancement would substantially enhance FMD prevention and control efforts in the country. Implementing these strategies together can mitigate the impact of FMD and protect the livestock industry in Algeria, thus contributing to regional and international FMD control efforts.

## Authors’ Contributions

MG, MA, MD, and RB: Played essential roles in collecting official statistics and analyzing official records. MG, MA, and MD: Initiated and guided the conceptualization of the study, conducted extensive literature search, and data compilation. MG and RB: Analyzed the data and drafted the manuscript with substantial input and revisions from all authors. All authors have read, reviewed, and approved the manuscript.
